# Aminoacyl‐tRNA synthetases in Charcot–Marie–Tooth disease: A gain or a loss?

**DOI:** 10.1111/jnc.15249

**Published:** 2020-12-19

**Authors:** Han Zhang, Zhong‐Wei Zhou, Litao Sun

**Affiliations:** ^1^ Institute of Medical Biology Chinese Academy of Medical Sciences and Peking Union Medical College Kunming China; ^2^ School of Medicine Sun Yat‐sen University Guangzhou China; ^3^ School of Public Health (Shenzhen) Sun Yat‐sen University Guangzhou China

**Keywords:** aminoacyl‐tRNA synthetases, animal model, charcot‐marie‐tooth disease, mutation, pathogenesis

## Abstract

Charcot‐Marie‐Tooth disease (CMT) is one of the most common inherited neurodegenerative disorders with an increasing number of CMT‐associated variants identified as causative factors, however, there has been no effective therapy for CMT to date. Aminoacyl‐tRNA synthetases (aaRS) are essential enzymes in translation by charging amino acids onto their cognate tRNAs during protein synthesis. Dominant monoallelic variants of aaRSs have been largely implicated in CMT. Some aaRSs variants affect enzymatic activity, demonstrating a loss‐of‐function property. In contrast, loss of aminoacylation activity is neither necessary nor sufficient for some aaRSs variants to cause CMT. Instead, accumulating evidence from CMT patient samples, animal genetic studies or protein conformational analysis has pinpointed toxic gain‐of‐function of aaRSs variants in CMT, suggesting complicated mechanisms underlying the pathogenesis of CMT. In this review, we summarize the latest advances in studies on CMT‐linked aaRSs, with a particular focus on their functions. The current challenges, future direction and the promising candidates for potential treatment of CMT are also discussed.

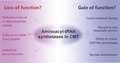

## Graphical abstract

Variants of some aminoacyl‐tRNA synthetases (aaRS) are strongly associated with Charcot–Marie–Tooth disease (CMT), one of the most common inherited neuromuscular disorders. Some variants affect enzymatic activity, showing loss‐of‐function effects. In contrast, evidence from CMT patient samples, animal genetic studies or protein conformational analysis has confirmed toxic gain‐of‐function of some aaRSs variants in CMT outside of aminoacylation. In this review, we will discuss the latest advances in studies on CMT‐associated aaRSs, with a particular focus on the complicated mechanisms in the pathogenesis of CMT.

**Abbreviations**
aaRS(s): aminoacyl‐tRNA synthetase(s)
AlaRS: alanyl‐tRNA synthetase
CMT: Charcot–Marie–Tooth disease
CMT1: demyelinating CMT type 1
CMT2: axonal CMT type 2
CMT2N: CMT type 2N
CMT2U: CMT type 2U
CMT2W: CMT type 2W
dHMN: distal hereditary motor neuronopathy
dHMN‐V: dHMN type V
DI‐CMT: dominant intermediate CMT
DI‐CMTC: DI‐CMT type C
EMAPII: endothelial monocyte‐activating polypeptide II
EPRS: glutamyl‐prolyl‐tRNA synthetase
GlyRS: glycyl‐tRNA synthetase
HDAC6: histone deacetylase 6
HDX: hydrogen‐deuterium exchange
HisRS: histidyl‐tRNA synthetase
iNPCs: induced neuronal progenitor cells
MetRS: methionyl‐tRNA synthetase
MS: mass spectrometry
MSC: multi‐synthetase complex
Nrp1: neuropilin‐1
PBMCs: peripheral blood mononuclear cells
SAXS: small‐angle X‐ray scattering
Trk: tropomyosin receptor kinase
TrkR(s): Trk receptor(s)
TrpRS: tryptophanyl‐tRNA synthetase
TyrRS: tyrosyl‐tRNA synthetase
VE‐cadherin: vascular endothelial‐cadherin
VEGF: vascular endothelial growth factor
WT: wild type

## 1 INTRODUCTION

Charcot–Marie–Tooth disease (CMT) is one of the most common inherited neuromuscular disorders with an estimated prevalence of 1/2,500 worldwide (Rossor et al., 2016; Skre, 1974). The major clinical manifestation of CMT is the degeneration of both motor and sensory peripheral nerves, leading to a loss of muscle tissue and touch sensation in bodily extremities (Patzko & Shy, 2011).

Based on electrophysiological and histopathological criteria, CMT is divided into two major groups: demyelinating CMT type 1 (CMT1) and axonal CMT type 2 (CMT2). CMT1 is the most prevalent type presented as demyelinating peripheral neuropathy, which manifests as markedly decreased nerve‐conduction velocities. In contrast, CMT2 accounts for approximately 20% of cases characterized by pathological axonal loss at nerve biopsy and has relatively normal conduction velocities (Pareyson & Marchesi, 2009). Beyond this clear classification, rapidly increasing knowledge of this disease has led to the recognition of other forms of CMT, such as a dominant intermediate CMT subtype (DI‐CMT) with features of both CMT1 and CMT2, and a pure motor form, distal hereditary motor neuronopathy (dHMN), characterized by the sparing of sensory nerves upon examination. Further subdivisions of these CMT types are mainly based on causative genes and variants.

In typical cases, CMT onset often occurs in the first two decades of life, and the disease processes slowly without affecting life expectancy (Laura et al., 2019; Pareyson & Marchesi, 2009). However, the age of onset, disease course and severity vary greatly based on the CMT subtype, causative genes and types of variants. Despite significant advances in the genetic diagnosis of CMT using next‐generation sequencing technology, no effective therapies have thus far been developed.

To date, over 100 CMT‐associated genes have been identified as causative factors (Laura et al., 2019). Among them, five genes (*GARS*, *YARS*, *AARS*, *HARS* and *WARS*) encode aminoacyl‐tRNA synthetases (aaRSs) including glycyl‐, tyrosyl‐, alanyl‐, histidyl‐ and tryptophanyl‐tRNA synthetases (GlyRS, TyrRS, AlaRS, HisRS and TrpRS, respectively) (Antonellis et al., 2003; Jordanova et al., 2006; Latour et al., 2010; Tsai et al., 2017; Vester et al., 2013), which is the largest family implicated in CMT and highlights the vital importance of aaRSs in the pathogenesis of CMT.

The aaRS family comprises ubiquitously expressed enzymes that are involved in the translation of the genetic code by charging amino acids onto their cognate tRNAs during protein synthesis (Ling et al., 2009). Based on structural features, 20 canonical aaRSs are divided into two classes that differ in the architecture of their active sites for adenylate synthesis. Class I aaRSs contain a typical Rossmann‐fold utilized for nucleotide binding and two well‐conserved signature sequences (HIGH and KMSKS), whereas class II aaRSs are less conserved and include three signature motifs (Motifs 1‐3) within a seven‐stranded β‐sheet and three flanking α‐helices. Among the five known CMT‐associated aaRSs, TryRS and TrpRS belong to class I, and the other three (GlyRS, AlaRS and HisRS) belong to class II.

Intriguingly, variants of these tRNA synthetase genes are strongly associated with CMT, but not all variants affect the aminoacylation activities of the enzymes. More strikingly, CMT‐like neuropathy phenotypes in animal models generated by some variants cannot be rescued by overexpression of wild type (WT) proteins, suggesting that CMT is very likely linked to toxic gain‐of‐function associated with the variants themselves. In this review, we discuss the latest advances in studies on aaRSs in CMT, particularly focusing on their roles in the pathogenesis of the disease.

## 2 *GARS* VARIANTS

**FIGURE 1:**
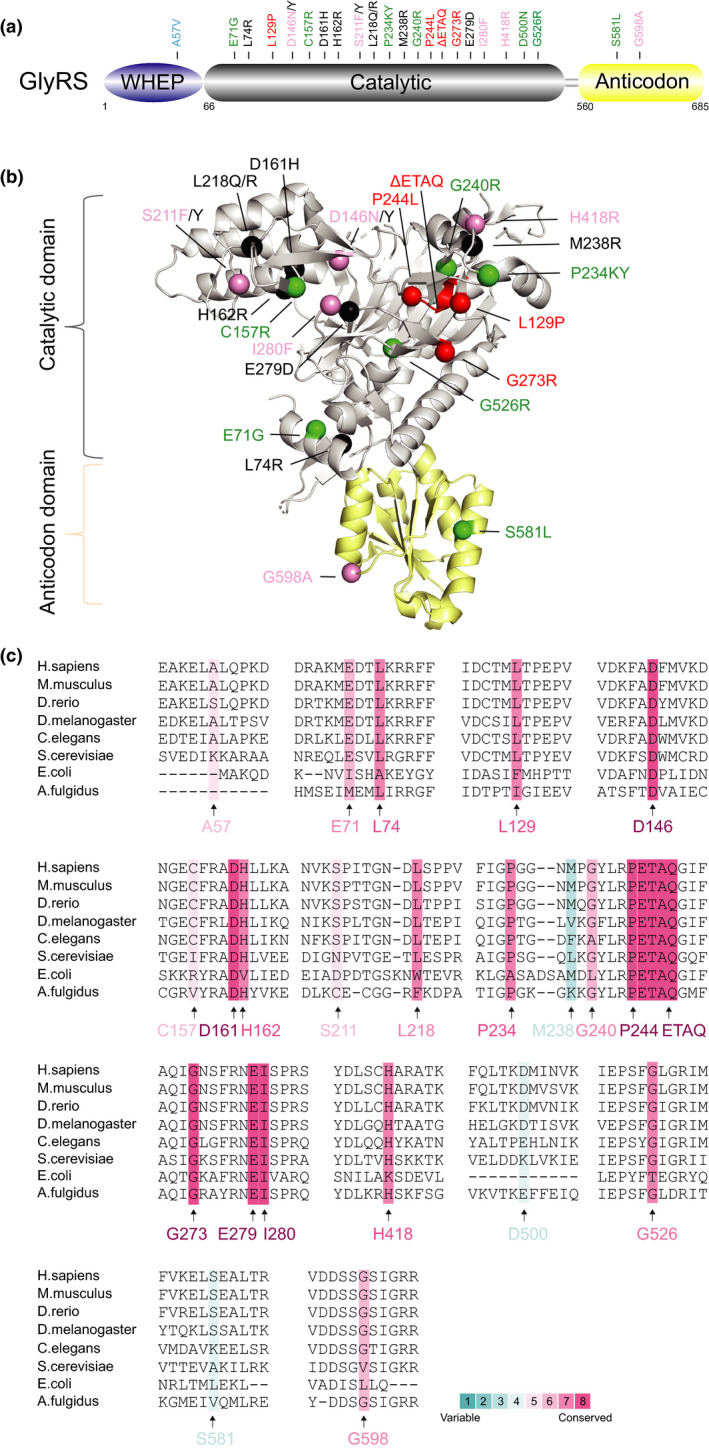
Distribution and conservation of CMT‐associated variant sites in human GlyRS. (a) Functional domains of GlyRS including a WHEP domain (in purple), a catalytic domain (in grey) and an anticodon domain (in yellow). (b) The crystal structure of human GlyRS (PDB entry 2PME). CMT variant sites in either schematic diagram (a) or crystal structure (b) are indicated with different colors based on enzymatic activity of each variant. CMT variants with WT‐like enzymatic activity (fully active) are colored in green; variants with the activity ≥1/2 are labeled in blue; variants with the activity <1/2 are indicated in pink; variants with no activity (inactive) are colored in red; variants with activity undetermined are indicated in black. The priority of enzymatic activity displayed here is ranked based on aminoacylation assays in patient sample > animal models > in vitro using purified human enzyme > yeast orthologs. (c) Evolutionary conservation analysis of CMT‐linked GlyRS across archaea, bacteria and eukaryotes. Sequence alignment of each GlyRS variant site is indicated by the color intensity, with blue representing variable and red representing conserved

GlyRS is a class II aaRS with a conserved catalytic domain composed of a central antiparallel β‐sheet flanked with α‐helices and three conserved sequence motifs (Xie et al., 2007), followed by an anticodon domain. The N‐terminal extension contains a specific appended helix‐turn‐helix structural motif named the WHEP domain, which derives from three of the five WHEP‐containing proteins TrpRS, HisRS and EPRS (Guo et al., 2010) (Figure 1a). GlyRS was the first tRNA synthetase implicated in CMT (Antonellis et al., 2003). To date, more than twenty missense variants of *GARS* have been discovered in patients with CMT2D (OMIM# 601472), an autosomal‐dominant axonal subtype of CMT, or dHMN type V (dHMN‐V, OMIM# 600794), a subtype of dHMN with upper‐limb predominance (Motley et al., 2010) (Table 1). Among them, most variants are located in the catalytic domain, one variant is in the WHEP domain and the remaining two are in the anticodon domain (Figure 1a and b). Interestingly, several variants (*GARS* ^E71G^, *GARS* ^L129P^, *GARS* ^G240R^, *GARS* ^I280F^, *GARS* ^H418R^, *GARS* ^G526R^ and *GARS* ^G598A^) are closely associated with the disease; however, some affect aminoacylation activity, while others do not (Griffin et al., 2014) (Table 1). For example, *GARS* ^E71G^ segregates with CMT2D in large families but has WT‐like aminoacylation activity (Antonellis et al., 2003, 2006; Nangle et al., 2007; Niehues et al., 2015). Given the differential enzymatic activities of GlyRS variants, it is not surprising for scientists to seek out other disease‐associated functions of these variants that are distinct from the canonical aminoacylation functions.

**TABLE 1:** Effect of CMT variants on aminoacylation activity and conformational change of aaRSs

*aaRS*	Variants	Segregation*	Age at onset	Aminoacylation activity**	Phenotype		Conformational change	References
					Yeast†	Animal model‡		
*GARS*	A57V	P1 in F1	12 y	*k*_cat_/*K*_m_: 1/2 (tRNA)	NA	ND	ND	(Griffin et al., 2014; Rohkamm et al., 2007)
	E71G	P17 in F1	17.7 y^b^	Normal (*Drosophila*); Normal (in vitro)	Viable	Neuronal phenotypes [trans‐OE, *Drosophila*]	Little change versus. WT	(Antonellis et al., 2003, 2006; Nangle et al., 2007; Niehues et al., 2015; Qin et al., 2014; Sivakumar et al., 2005)
	L74R	P6 in F1	13.3 y^b^	ND	ND	ND	ND	(Yu et al., 2018)
	L129P	P5 in F1	16.9 y^b^	Loss (in vitro)	Reduced	ND	HDX increase^d,e,f^: 37%	(Antonellis et al., 2003, 2006; He et al., 2011; Nangle et al., 2007; Sivakumar et al., 2005)
	D146N	P2 in F1	15 y^b^	*k*_cat_/*K*_m_: 1/14 (tRNA)	Reduced	ND	ND	(Griffin et al., 2014; Lee et al., 2012)
	D146Y	P1 in F1	3 mo	ND	ND	ND	ND	(Liao et al., 2015)
	C157R /C201R M	‐‐	‐‐	Insignificant reduced (± mouse); Reduced (+/+mouse)	ND	Neuropathy phenotypes in ±; postnatal death in +/+ [KI, mouse]	ND	(Achilli et al., 2009)
	D161H	P3 in F1	17 y^c^	ND	ND	ND	ND	(Nan et al., 2019)
	H162R /H218R Mt	P2 in F1	Early 20s	ND	ND	ND	ND	(Boczonadi et al., 2018)
	S211F	P4 in F2 from 2C	8 y^c^	*k*_cat_/*K*_m_: 1/4,400 (tRNA)	NA	ND	ND	(Griffin et al., 2014; Lee et al., 2012; Sun et al., 2017)
	S211Y	P2 in F1	11 y^c^	ND	ND	ND	ND	(Yalcouye et al., 2019)
	L218Q	P1 in F1	Toddler	ND	ND	ND	ND	(Kawakami et al., 2014)
	L218R	P4 in F1	8.7 mo^c^	ND	ND	ND	ND	(Chung et al., 2018)
	P234KY /P278KY M	–	–	Insignificant reduced (±mouse); Normal (in vitro) *k*_cat_/*K*_m_: 1/500 (tRNA)	Viable or Lethal	Neuropathy phenotypes in ±; embryonic lethal in +/+ [KI, mouse]; Neuronal phenotypes [trans‐OE, *Drosophila*]	HDX increase^d,e,f^: 15%	(Grice et al., 2015; He et al., 2015; Morelli et al., 2019; Nangle et al., 2007; Seburn et al., 2006; Stum et al., 2011)
	M238R	P1 in F1	2 y	ND	ND	ND	ND	(Liao et al., 2015)
	G240R	P8 in F1^a^	23 y^b^	Normal (*Drosophila*); Loss (in vitro); *k*_cat_/*K*_m_: 1/110 (tRNA)	Viable	Neuronal phenotypes [trans‐OE, *Drosophila*]	HDX increase^d,e,f^: 30%	(Antonellis et al., 2003, 2006; He et al., 2011; Niehues et al., 2015; Sivakumar et al., 2005)
	P244L	P1 in F1	Adolescence	*k*_cat_/*K*_m_: undetectable	Lethal	ND	ND	(Abe & Hayasaka, 2009; Griffin et al., 2014)
	ΔETAQ	P1 in F1	13 mo	*k*_cat_/*K*_m_: 1/11000 (tRNA)	Lethal	Neuropathy phenotypes in ± [microinj, mouse]	ND	(Morelli et al., 2019)
	G273R	P2 in F2	11.5 y^b^	ND	Lethal	ND	ND	(Lee et al., 2019)
	E279D	P8 in F1	Adolescence	ND	ND	ND	ND	(Sun et al., 2015)
	I280F	P4 in F2 from 2C	22.5 y^b^	*k*_cat_/*K*_m_: 1/1,700 (tRNA)	Viable	ND	ND	(Griffin et al., 2014; James et al., 2006; Klein et al., 2014)
	H418R	P8 in F1	26 y^b^	Reduced (in vitro); *k*_cat_/*K*_m_: 1/16 (tRNA)	Lethal	ND	ND	(Antonellis et al., 2006; Griffin et al., 2014; Nangle et al., 2007; Sivakumar et al., 2005)
	D500N	P6 in F1	21.7 y^b^	Normal (in vitro)	NA	ND	ND	(Del Bo et al., 2006; Griffin et al., 2014; Nangle et al., 2007)
	G526R	P18 in F4 from 2C	21.4 y^c^	Normal (*Drosophila*); Loss (in vitro); *k*_cat_/*K*_m_: 1/44,000 (tRNA)	Lethal	Neuronal phenotypes [trans‐OE, *Drosophila*]	Little change versus. WT; *R_g_* ^g^: 15.6%; HDX increase^d,e,f^: 16%	(Antonellis et al., 2003, 2006; Dubourg et al., 2006; He et al., 2011; Niehues et al., 2015; Xie et al., 2007)
	S581L	P1 in F1	4 y	Normal (in vitro); *k*_cat_/*K*_m_: 1/2 (tRNA)	NA	ND	Little change versus. WT; HDX increase^d,e,f^: 18%	(Cader et al., 2007; Griffin et al., 2014; He et al., 2011; James et al., 2006; Nangle et al., 2007)
	G598A	P3 in F2 from 2C	Infantile	*k*_cat_/*K*_m_: 1/180 (tRNA)	Viable	ND	HDX increase^d,e,f^: 22%	(Eskuri et al., 2012; Griffin et al., 2014; He et al., 2011; James et al., 2006; Stum et al., 2011)
*YARS*	G41R	P15 in F1	1st and 2nd decades	Reduced (*Drosophila*); Loss (in vitro)	Lethal	Neuronal phenotypes [trans‐OE, *Drosophila*]	*R_g_*: 5.2% R_H_ ^h^: 6.9% HDX increase^d,e,f^: 1.3%	(Gonzaga‐Jauregui et al., 2015; Jordanova et al., 2003, 2006; Storkebaum et al., 2009)
	D81I	P1 in F1	23 y	ND	ND	ND	ND	(Hyun et al., 2014)
	Δ153−156	P1 in F1	ND	Reduced (*Drosophila*); Reduced (in vitro)	Reduced	Neuronal phenotypes [trans‐OE, *Drosophila*]	*R_g_*: −2.4% R_H_: −13.8% HDX increase^d,e,f^: none	(Jordanova et al., 2006; Storkebaum et al., 2009)
	E196K	P38 in F1	7–59 y	Normal (*Drosophila*); Normal (in vitro)	Viable	Neuronal phenotypes [trans‐OE, *Drosophila*]	*R_g_*: 5.8% R_H_: 10.3% HDX increase^d,e,f^: 10.8%	(Froelich & First, 2011; Gonzaga‐Jauregui et al., 2015; Jordanova et al., 2003, 2006; Storkebaum et al., 2009)
	E196Q	P7 in F1	ND	ND	Reduced	ND	ND	(Gonzaga‐Jauregui et al., 2015)
*AARS*	N71Y	P7 in F1	31.5 y^c^	Loss (in vitro); *k*_cat_/*K*_m_: 1/4,130 (tRNA)	Lethal	ND	ND	(Lin et al., 2011; McLaughlin et al., 2012)
	G102R	P5 in F1	34 y^c^	ND	Lethal	ND	ND	(Motley et al., 2015)
	R326W	P11 in F1	35.8 y^c^	ND	Lethal	Abnormal embryos [microinj, Zebrafish]	ND	(Weterman et al., 2018)
	R329H	P66 in F8 from 4C	25.0 y^c^	Loss (in vitro); *k*_cat_/*K*_m_: 1/50 (tRNA)	Lethal	ND	ND	(Bansagi et al., 2015; Latour et al., 2010; McLaughlin et al., 2012)
	R337K	P6 in F1	19.7 y^c^	*k*_cat_/*K*_m_: 3.87 (tRNA)	Enhanced	Abnormal embryos [microinj, Zebrafish]	ND	(McLaughlin et al., 2012; Weterman et al., 2018)
	S627L	P4 in F1	30 y^c^	ND	Reduced	Abnormal embryos [microinj, Zebrafish]	ND	(Weterman et al., 2018)
	E688G	P3 in F1	Early onset	ND	ND	ND	ND	(Bansagi et al., 2015)
	E778A	P4 in F1	ND	Normal (in vitro); *k*_cat_/*K*_m_: 1/1.4 (tRNA)	Viable	ND	ND	(McLaughlin et al., 2012)
	D893N	P4 in F1	28.5 y^c^	ND	ND	ND	ND	(Zhao et al., 2012)
*HARS*	T132I	P10 in F1	23.7 y^c^	Loss (in vitro); *k*_cat_/*K*_m_: undetectable	Lethal	ND	*R_g_*: 2.3% D_H_ ^h^: 4% HDX decrease^d,e,f^: 4%	(Blocquel et al., 2019; Safka Brozkova et al., 2015)
	P134H	P5 in F1	Childhood	Normal (patient); Reduced (in vitro); *k*_cat_/*K*_m_: 1/83 (His); 1/25 (ATP)	Lethal	ND	*R_g_*: 5.2% D_H_: 18% HDX increase^d,e,f^: 6%	(Blocquel et al., 2019; Safka Brozkova et al., 2015)
	R137Q	P1 in F1	49 y	ND	Lethal	Neurotoxicity [trans‐OE, *C. elegans*]	ND	(Vester et al., 2013)
	V155G	P5 in F1	2nd decade	Reduced (in vitro); *k*_cat_/*K*_m_: 1/2.2 (tRNA); 1/166.7 (His); 1/342.1 (ATP)	Viable	ND	ND	(Abbott et al., 2018)
	D175E	P4 in F1	25.5 y^c^	Reduced (in vitro); *k*_cat_/*K*_m_: 1/2,000 (His); 1/62 (ATP)	Reduced	ND	*R_g_*: 2.3% D_H_: 20% HDX decrease^d,e,f^: 5%	(Blocquel et al., 2019; Safka Brozkova et al., 2015)
	Y330C	P2 in F1	Childhood	Reduced (in vitro); *k*_cat_/*K*_m_: 1/4.1 (tRNA); 1/125 (His); 1/477.9 (ATP)	Reduced	ND	ND	(Abbott et al., 2018)
	S356N	P1 in F1	10 y	Reduced (in vitro); *k*_cat_/*K*_m_: 1/4.6 (tRNA); 1/12.5 (His); 1/866.7 (ATP)	Reduced	ND	ND	(Abbott et al., 2018)
	D364Y	P9 in F1	21.9 y^c^	Normal (in vitro); *k*_cat_/*K*_m_: 1/50 (His); 1/1.2 (ATP)	Lethal	Neurotoxicity [trans‐OE, *C. elegans*]	*R_g_*: 4.1% D_H_: 16% HDX increase^d,e,f^: 3%	(Blocquel et al., 2019; Safka Brozkova et al., 2015)
*WARS*	H257R	P12 in F3 from 2C	11.8 y^c^	Reduced (in vitro)	Enhanced^††^	ND	ND	(Tsai et al., 2017)
	D314G	P5 in F1	17.8 y^b^	ND	ND	ND	ND	(Wang et al., 2019)
*MARS*	A397T	P1 in F1	< 1 y	ND	Lethal	ND	ND	(Gillespie et al., 2019)
	R618C	P3 in F1	56 y^c^	ND	Lethal	ND	ND	(Gonzalez et al., 2013)
	R737W	P2 in F1	12 y^c^	ND	ND	ND	ND	(Sagi‐Dain et al., 2018)
	P800T	P5 in F3 from 2C	34.3 y^c^	ND	ND	ND	ND	(Hirano et al., 2016; Hyun et al., 2014; Nam et al., 2016)

Note

M, mouse; +/+, homozygous; ±, heterozygous; Mt, mitochondria; His, histidine; versus. versus; WT, wild type; Ref., references; ND, not determined.

^a^ This variant was identified in 2 families with information in one family unknown.

^b^ Average age at onset.

^c^ Average age at onset of available individual(s) except those deceased individuals, asymptomatic individuals, affected individuals with age at onset unknown or ambiguous, or alive individuals but refused to have a clinical evaluation.

^g^ Difference of radius of gyration (*R_g_*, Å) for each variant relative to WT enzyme based on SAXS analysis. The value is calculated using the formula of (*R_g_* ^Vriant^‐*R_g_* ^WT^)/*R_g_* ^WT^ × 100%, with a positive value indicating size increase and a negative value indicating size decrease, respectively.

^h^ Difference of hydrodynamic radius (R_H_, nm) or diameter (D_H_, nm) of each variant relative to WT enzyme based on the switchSENSE^®^ Technology. The value is calculated using the formula of (R_H_ or D_H_^Variant^‐ R_H_ or D_H_^WT^)/R_H_ or D_H_^WT^ × 100%, with a positive value indicating a larger protein size and a negative value indicating a smaller protein size, respectively.

* Family segregation of genetic variants. P, person(s) carrying the variant; F, family(ies); C, countries.

** Aminoacylation activity of each variant is ranked based on different assays from in vivo to in vitro charging to in vitro kinetics. In in vivo assays, the types of models are shown in parentheses. Enzymatic activity in both in vivo and in vitro assays is shown with ‘Normal’ indicating WT‐like activity, ‘Loss’ indicating inactive, and ‘Reduced’ indicating activity between ‘Normal’ and ‘Loss’. For kinetic analysis, catalytic rate constant/Michaelis constant (*k*_cat_/*K*_m_) is shown in each variant relative to WT for different substrates including tRNA, amino acid or ATP shown in parentheses; ‘undetectable’ indicates values that are below the limits of kinetic detection.

^†^ Growth of yeast strain containing variant form compared with WT strain. NA, not applicable because of the variant that could not be modeled in yeast ortholog.

^††^ Growth of yeast strains containing human variant relative to human WT gene.

^‡^ Phenotypes of each variant in animal models with the genetic manipulation of species shown in square brackets. trans‐OE, transgenic over‐expression; KI, knockin; microinj, microinjection.

^d,e,f^ Average increase/decrease in deuterium incorporation for each variant relative to WT enzyme after 1 h^d^ or at 1,000 s^e^ or after 10,000 s^f^ of exchange based on HDX analysis. h, hour; s, seconds.

### 2.1 Animal models of GlyRS‐linked CMT

Among all CMT‐associated aaRSs, GlyRS is well‐established and the most studied in animal models. In *Drosophila* models, GlyRS variants significantly reduce the levels of newly synthesized proteins, and this translational defect is not attributed to altered tRNA aminoacylation because overexpression of *Drosophila* gars fails to rescue impaired protein translation (Niehues et al., 2015). Additionally, the enzymatic activity in the tissues of mice carrying heterozygous *Gars* ^Nmf249/+^ and *Gars* ^C201R/+^ variants (corresponding to *GARS* ^P234KY^ and *GARS* ^C157R^ in human) was not significantly decreased compared with that in WT animals (Achilli et al., 2009; Seburn et al., 2006). Notably, overexpression of WT GlyRS could not improve the neuropathy phenotypes in either *Gars* ^Nmf249/+^ or *Gars* ^C201R/+^ mice (Motley et al., 2011), and the same conclusion regarding another *GARS* ^G240R^ variant was reached with *Drosophila* CMT models, which showed no phenotypic rescue upon overexpression of the WT proteins (Niehues et al., 2015).

Recently, one group identified a de novo *GARS* variant (*GARS* ^ΔETAQ^) in a single patient with severe peripheral neuropathy, and this allele was further introduced into a mouse model (Morelli et al., 2019). This mouse model carrying a human disease allele displays primary features of peripheral neuropathy. Strikingly, the allele‐specific knockdown of *GARS* ^ΔETAQ^ using RNAi prevents the neuropathy phenotypes in mice, and the same efficacy is confirmed in *Gars* ^Nmf249/+^ mice, both before and after onset. These findings provide important proof‐of‐concept for virally delivered RNAi‐based gene therapy for treating CMT2D; however, whether this approach can be applied to other CMT‐causing single‐base pair variants requires additional research. Of note, in this study, re‐evaluation of in vitro kinetic properties and yeast models for the P234KY allele supported a loss‐of‐function effect (Morelli et al., 2019), contrary to previous reports (Table 1) (Nangle et al., 2007; Seburn et al., 2006; Stum et al., 2011). Such discrepancies in activity assays may reflect different sensitivities of experimental settings.

Given the well‐characterized *Gars* ^Nmf249/+^ in mice, one group further modeled the P234KY variant in *Drosophila* (*gars* ^P234KY^). Ubiquitous expression of *gars* ^P234KY^ was shown to severely affect fly fitness, with no adults emerging, whereas pan‐neuronal expression caused late pupal lethality, suggesting that toxicity may manifest or derive from tissues beyond the nervous system (Ermanoska et al., 2014). Indeed, muscular expression of *gars* ^P234KY^ in flies induces significant neuronal defects, and the same is true for the *gars* ^G240R^ variant, suggesting that the pathology has a noncell autonomous contribution (Grice et al., 2015). Interestingly, the neuronal toxicity of *gars* ^P234KY^ is dependent on the WHEP domain since its removal from *gars* ^P234KY^ abrogates neuromuscular and survival defects, revealing a clear dominant toxic gain‐of‐function mechanism for variants that may contribute to the pathology of CMT2D (Grice et al., 2015).

In contrast, mice carrying homozygous *GARS* variants, such as those of *Gars* ^Nmf249/Nmf249^ and *Gars* ^C201R/C201R^, are not viable or even undergo early death, and perinatal lethality can be rescued by overexpression of the WT proteins (Achilli et al., 2009; Seburn et al., 2006). Likewise, a homozygous *gars* variant (*gars* ^P98L^) in *Drosophila*, although not the cause of CMT, leads to defects in dendritic and axonal terminal arborization, which can be fully rescued by transgenic expression of WT human *GARS* (Chihara et al., 2007). This finding provides evidence that human GlyRS has equivalent functions in *Drosophila*. However, the defects in fly models could be only partially rescued by the human *GARS* ^E71G^ variant, whereas the *GARS* ^L129P^ variant did not show any rescue capability, indicating that human CMT2D variants do have loss‐of‐function properties and may affect the normal function of GlyRS to different degrees. Thus, based on studies from animal models, different *GARS* variants may cause the same disease via distinct mechanisms, with some variants having toxic gain‐of‐function effects and others demonstrating undefined loss‐of‐function properties.

### 2.2 Conformational changes in CMT‐linked GARS variants

To date, crystal structures of three *GARS* variants, *GARS* ^G526R^, *GARS* ^S581L^ and *GARS* ^E71G^, have been solved; however, few conformational changes were observed compared with the WT proteins (Cader et al., 2007; Qin et al., 2014; Xie et al., 2007) (Table 1). Nonetheless, different CMT‐causing variants have distinct effects on dimer formation. For example, *GARS* ^D500N^, *GARS* ^G526R^ and *GARS* ^S581L^ strengthen the capacity of dimer formation, whereas *GARS* ^L129P^ and *GARS* ^G240R^ disrupt dimer formation (Nangle et al., 2007). Because the dimeric form of GlyRS is essential for aminoacylation, both *GARS* ^L129P^ and *GARS* ^G240R^ show a loss of enzyme activity, while *GARS* ^D500N^ and *GARS* ^S581L^ demonstrate full aminoacylation activity compared to that of the WT enzyme. However, despite an enhanced dimer formation capability, *GARS* ^G526R^ has abolished aminoacylation activity (Nangle et al., 2007). Therefore, considering this together with another fully active variant (*GARS* ^E71G^), almost half of these CMT‐causing variants are active, further supporting the conclusion that CMT is not simply caused by a deficiency in aminoacylation. Furthermore, this finding also raises the possibility that CMT‐causing variants may disrupt an unknown function of GlyRS, leading to a toxic gain‐of‐function that is associated with only GlyRS variants.

Considering these data, the same research group further explored five *GARS* variants (*GARS* ^L129P^, *GARS* ^G240R^, *GARS* ^G526R^, *GARS* ^S581L^ and *GARS* ^G598A^) in solution utilizing hydrogen‐deuterium exchange (HDX) analysis monitored by mass spectrometry (MS) (He et al., 2011). Interestingly, despite their differential effects on dimerization and aminoacylation activity (Griffin et al., 2014; Nangle et al., 2007), all five variants exhibit the same neomorphic conformational opening that is mostly buried in WT GlyRS (He et al., 2011). This conformational change also occurs in the mouse carrying the *GARS* ^P234KY^ variant, which has a similar neomorphic structural opening. Further small‐angle X‐ray scattering (SAXS) analysis of the *GARS* ^G526R^ variant confirmed that this neomorphic structural opening is associated with an unknown physiological function of GlyRS that is suppressed by the WHEP domain. Perhaps for this reason, these CMT‐causing variants might disrupt WHEP‐mediated suppression, resulting in gain‐of‐function phenotypes. Whether the induced conformational changes confer the variants with the ability to interact with other proteins needs to be further addressed.

It is important to note that, based on conservation analysis (Figure 1c), some variant sites such as E71 and G526 in *GARS*, show less divergence in the evolutionary progression to humans, suggesting essential roles of these sites during evolution; however, based on the above findings, variations in these sites do not significantly affect enzymatic activity, further supporting non‐canonical activities outside of aminoacylation imparted by these variants. Other essential functions of aaRSs beyond translation have been well documented (Guo & Schimmel, 2013; Guo et al., 2010).

### 2.3 Interaction partners of GARS variants

In fact, several interaction partners of *GARS* variants as well as the molecular mechanisms underlying the interactions have been uncovered. Variant, but not WT, GlyRS are capable of binding the neuropilin‐1 (Nrp1) receptor, and such an aberrant interaction competitively interferes with the binding of the cognate ligand vascular endothelial growth factor (VEGF) to Nrp1, thereby contributing to motor defects in CMT2D (He et al., 2015). More importantly, aberrant signaling of GlyRS/Nrp1/VEGF is observable in the neural tissues of *Gars* ^Nmf249/+^ mice, and VEGF overexpression can partially rescue motor defects in *Gars* ^Nmf249/+^ mice. The same GlyRS‐Nrp1 interaction also occurs in lymphocytes from CMT2D patients with the *GARS* ^L129P^ variant. This work demonstrates that CMT arises from the neomorphic activity of misfolded GlyRS interacting with susceptible signaling targets independent of aminoacylation. Nonetheless, not all GlyRS variants contribute to the disease through the GlyRS‐Nrp1 interaction. For example, the *GARS* ^ΔETAQ^ variant does not have a strong interaction with Nrp1, suggesting distinct mechanisms for different variants (Morelli et al., 2019). The possibility of Nrp1‐interacting GlyRS variants interacting with other extracellular and/or intercellular targets cannot be ruled out.

In addition to Nrp1, tropomyosin receptor kinase (Trk) receptors (TrkRs) were subsequently shown to interact with CMT2D‐linked GlyRS variants (*GARS* ^CMT2D^). Unlike Nrp1/VEGF signaling, which targets motor axons, TrkR signaling specifically acts on sensory axons. Variant, but not WT, GlyRS misactivates Trk signaling by binding to multiple TrkRs, leading to defective differentiation and development of sensory neurons (Sleigh et al., 2017). Interestingly, *Gars* ^C201R/+^ mice exhibited nonprogressive perturbation of sensory neuronal fate during early stages of development, in line with the clinical features of some CMT2D patients who present subtle and undiagnosed sensory symptoms in advance of motor deficits (Sleigh et al., 2017). This finding also explains the absence of sensory defects in patients with dHMN‐V who present predominantly with motor degeneration.

The aberrant interaction of GlyRS with Nrp1 or TrkR depends on the extracellular domains of Nrp1 or TrkR. One intracellular partner, histone deacetylase 6 (HDAC6), was recently identified and surprisingly shown to aberrantly interact with almost all *GARS* ^CMT2D^ variants in reach (*GARS* ^E71G^, *GARS* ^L129P^, *GARS* ^S211F^, *GARS* ^G240R^, *GARS* ^E279D^, *GARS* ^H418R^, *GARS* ^G526R^, *GARS* ^S581L^ and *GARS* ^G598A^), and this aberrant crosstalk was shown to functionally stimulate the deacetylase activity of HDAC6 on α‐tubulin (Mo et al., 2018). Deacetylation of α‐tubulin further causes axonal transport deficits prior to disease onset in *Gars* ^Nmf249/+^ mice, with specific targeting at peripheral nerves rather than the brain or spinal cord. More essentially, the defective phenotypes can be rescued by the HDAC6 inhibitor, and the same is true for *Gars* ^C201R/+^ mice from another study (Benoy et al., 2018). Although abnormal GlyRS‐HDAC6 interactions have been identified in many human *GARS* ^CMT2D^ variants, the differential degrees of interplay among variants is closely associated with distinct clinical manifestations among CMT2D patients. For example, the *GARS* ^S581L^ and *GARS* ^G598A^ variants trigger the strongest HDAC6 interaction among all variants, of which the *GARS* ^G598A^ variant can also interact with Nrp1, concordant with unfavorable clinical features of infantile onset and extreme severity for patients harboring the *GARS* ^G598A^ variant (Mo et al., 2018). For the other human *GARS* ^CMT2D^ variants with relatively weak HDAC6 interactions, the possibility of alternative pathogenic mechanisms in CMT2D cannot be excluded.

### 2.4 Mitochondrial role of GlyRS in CMT

It is worth noting that GlyRS is one of two aaRSs (the other is lysyl‐tRNA synthetase) encoding both cytosolic and mitochondrial forms; however, little is known about the mitochondrial role of GlyRS and how it affects the phenotypes of diseases such as CMT.

A recent study identified a novel disease‐associated dominant variant of *GARS* ^H162R^ (reported as *GARS* ^H216R^ because of inclusion of the mitochondrial target sequence) in patients presenting with typical clinical and electrophysiological signs of dHMN‐V (Boczonadi et al., 2018). Indeed, GlyRS is present in RNA granules in mitochondria and is involved in mitochondrial translation; downregulation of *GARS* results in defective mitochondrial translation in neuronal cells and myoblasts but not in fibroblasts, indicating a tissue‐specific feature (Boczonadi et al., 2018). Interestingly, reduced mitochondrial respiration and decreased calcium uptake are found in induced neuronal progenitor cells (iNPCs) generated from patient carrying the *GARS* ^H162R^ variant, suggesting that this neuropathy‐associated variant leads to a complicated alteration of mitochondrial function in neurons. In *Gars* ^C201R^ mice, mitochondrial dysfunction is found in only the sciatic nerve, while the other five highly metabolic tissues display no mitochondrial defects, further supporting a tissue‐specific mechanism in vivo. This study, together with the complex interactions of GlyRS exemplified above, suggests that more cellular compartments are involved in aaRS‐linked CMT in addition to the cytoplasm and mitochondria.

## 3 *YARS* VARIANTS

**FIGURE 2:**
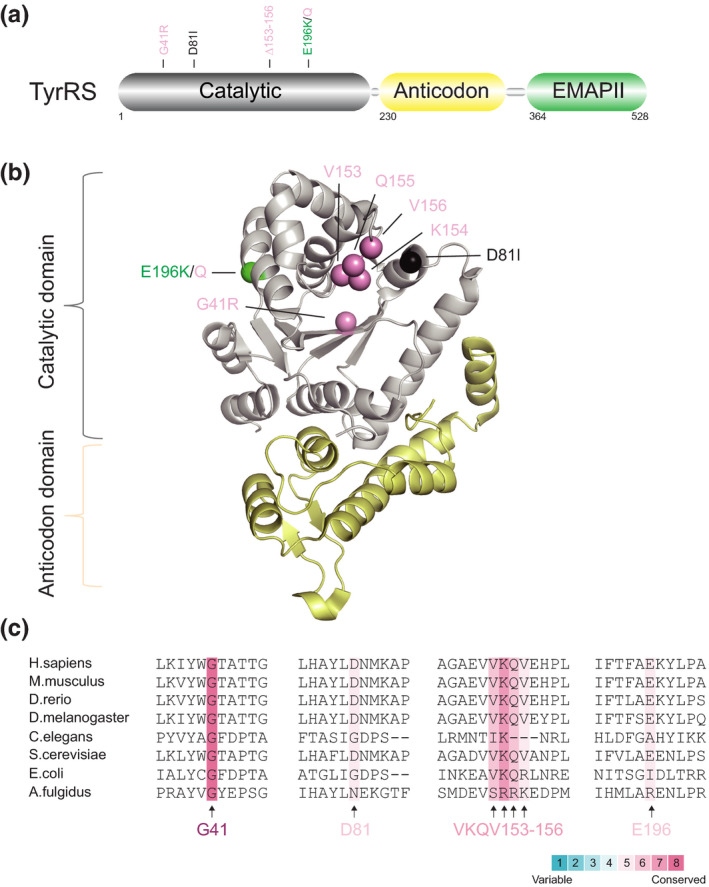
Distribution and conservation of CMT‐associated variant sites in human TyrRS. (a) Functional domains of TyrRS including a catalytic domain (in grey), an anticodon domain (in yellow) and an EMAPII domain (in green). (b) The crystal structure of human TyrRS without the C‐terminal EMAPII domain (PDB entry 1N3L). CMT variant sites in either schematic diagram (a) or crystal structure (b) are indicated with different colors based on enzymatic activity of each variant with the same priority ranking as Figure 1b. (c) Evolutionary conservation analysis of CMT‐linked TyrRS across species. Sequence alignment of each variant site is indicated by the color intensity, with blue representing variable and red representing conserved

Similar to other class I aaRSs, TyrRS has a Rossmann‐fold domain that consists of mostly parallel β‐strands connected by α‐helices as the catalytic domain (Blocquel et al., 2017); however, both TyrRS and TrpRS function as homodimers, while the remaining class I aaRSs are functional monomers. In addition to an N‐terminal catalytic domain and an anticodon domain, the C‐terminal fragment of human TyrRS is highly homologous to endothelial monocyte‐activating polypeptide II (EMAPII) (Kao et al., 1992), a known cytokine that is dispensable for aminoacylation by TyrRS (Figure 2a). To date, five catalytic domain variants of *YARS* have been reported in DI‐CMT type C (DI‐CMTC or CMTDIC, OMIM# 608323) (Gonzaga‐Jauregui et al., 2015; Hyun et al., 2014; Jordanova et al., 2006) (Table 1 and Figure 2a and b); three variants (*YARS* ^G41R^, *YARS* ^E196K^ and *YARS* ^E196Q^) segregate with DI‐CMTC in large families, whereas the remaining two variants (*YARS* ^D81I^ and *YARS* ^Δ153‐156^) have been found in only one patient each. Notably, the highly conserved *YARS* ^G41R^ and *YARS* ^Δ153‐156^ variants (Figure 2c) are defective in their ability to activate tyrosine, whereas the *YARS* ^E196K^ variant does not affect the formation of the tyrosyl‐adenylate intermediate but rather enhances the catalytic rate compared with that achieved with the WT enzyme (Froelich & First, 2011). In *Drosophila* models, the same conclusion of transgenic *YARS* ^E196K^ variant overexpression severely impairing motor performance but having normal enzymatic activity is reached (Storkebaum et al., 2009). In contrast, the *YARS* ^G41R^ and *YARS* ^Δ153‐156^ variants induce less toxicity in flies than the *YARS* ^E196K^ variant despite the severely decreased enzymatic activities of the *YARS* ^G41R^ and *YARS* ^Δ153‐156^ variants in *Drosophila*. Furthermore, *Drosophila* in which the *yars* is inhibited by 50% display normal motor performance. These findings indicate that the loss of aminoacylation activity is neither necessary nor sufficient to cause peripheral neuropathy, suggesting that TyrRS‐linked neurodegeneration results from a gain‐of‐function of TyrRS variants separate from aminoacylation.

To further pinpoint the functions gained from *YARS* variants, Xiang‐lei Yang and her colleagues uncovered conformational changes induced by the *YARS* ^G41R^ and *YARS* ^E196K^ variants; the *YARS* ^Δ153‐156^ variant did not induce conformational change but did expose the same area that was opened by the *YARS* ^G41R^ and *YARS* ^E196K^ variants (Blocquel et al., 2017). In addition, all three variants had the same enhanced binding affinity for TRIM28. These data suggest that DI‐CMTC, like CMT2D, is caused by a specific protein structure change that allows the generation of an alternative stable conformation for potential pathological interactions. The fact that the *YARS* ^Δ153‐156^ variant lacks conformational change but aberrantly interacts with TRIM28 suggests that residues 153–156 act as a structural blocker to prevent aberrant interactions of the WT enzyme (Blocquel et al., 2017).

Nevertheless, the abnormal TyrRS‐TRIM28 interaction should not be the only dysregulated pathway for DI‐CMTC variants since the TRIM28 ortholog is not found in *Drosophila*, suggesting alternative pathological mechanisms for CMT‐like phenotypes in fly models. Indeed, TyrRS can translocate to the nucleus for protection from DNA damage under oxidative stress, and this beneficial effect of nuclear TyrRS is achieved by activation of the transcription factor E2F1 (Wei et al., 2014). Further studies verified significant increases in the expression levels of E2F1‐regulated target genes in peripheral blood mononuclear cells (PBMCs) from patients with DI‐CMTC carrying the *YARS* ^G41R^ and *YARS* ^E196K^ variants (Bervoets et al., 2019). Remarkably, inhibition of nuclear TyrRS using pharmacological or genetic approaches suppresses the hallmark phenotypes of CMT in *Drosophila*, highlighting the importance of nuclear TyrRS variant for CMT neuropathology; however, the involvement of other cellular compartments or molecules mediating the toxicities outside the nucleus cannot be excluded.

Collectively, despite completely different molecular architectures for catalysis of class I and class II aaRSs (e.g., TyrRS and GlyRS), both types of architecture may predispose the enzymes to conformational perturbations that permit aberrant interactions. The nuclear involvement of TyrRS CMT neurotoxicity could also have essential implications for other CMT‐associated aaRSs.

## 4 *AARS* VARIANTS

**FIGURE 3:**
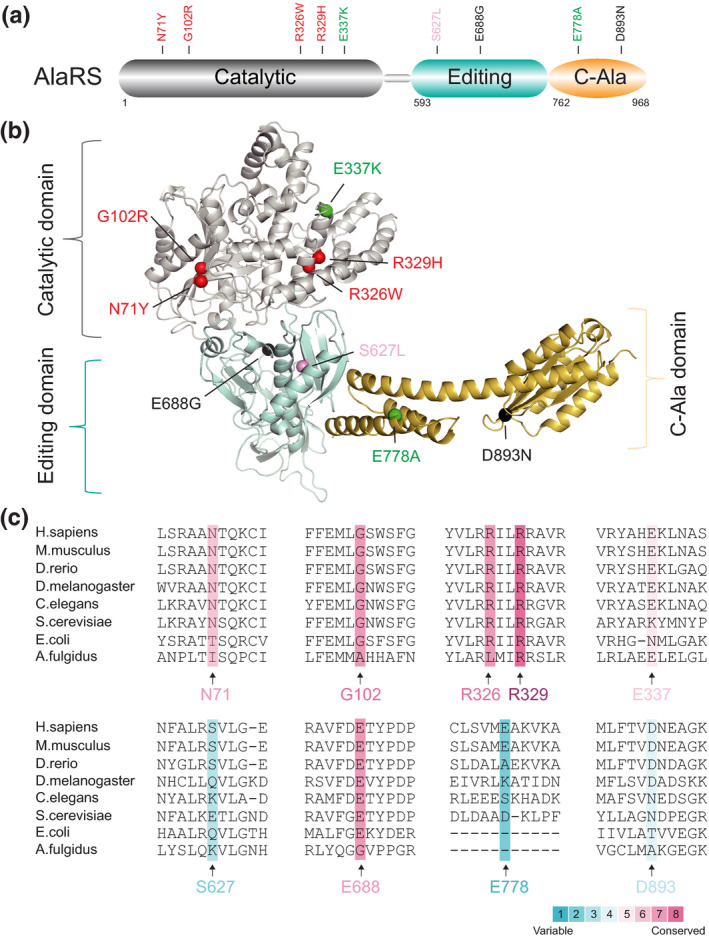
Distribution and conservation of CMT‐associated variant sites in human AlaRS. (a) Functional domains of AlaRS including a catalytic domain (in grey), an editing domain (in palegreen) and a C‐Ala domain (in orange). (b) The structure model of AlaRS was generated by the protein structure homology‐modelling server SWISS‐MODEL (Waterhouse et al., 2018). Human AlaRS catalytic domain (PDB entry 5KNN) and C‐Ala domain (PDB entry 5T5S) were further docked into the model and adjusted manually. CMT variant sites in either schematic diagram (a) or structure model (b) are indicated with different colors based on enzymatic activity of each variant with the same priority ranking as Figure 1b. (c) Evolutionary conservation analysis of CMT‐linked AlaRS across species. Sequence alignment of each variant site is indicated by the color intensity, with blue representing variable and red representing conserved

AlaRS is the third aaRS known to be involved in CMT. Unlike other CMT‐associated aaRSs, AlaRS does not have an anticodon domain but rather has an editing domain and a C‐terminal domain C‐Ala (Figure 3a). To date, nine *AARS* variants leading to dominant axonal CMT type 2N (CMT2N, OMIM# 613287) have been reported worldwide (Bansagi et al., 2015; Latour et al., 2010; Lin et al., 2011; McLaughlin et al., 2012; Motley et al., 2015; Weterman et al., 2018; Zhao et al., 2012) (Table 1). Among them, five variants (*AARS* ^N71Y^, *AARS* ^G102R^, *AARS* ^R326W^, *AARS* ^R329H^ and *AARS* ^R337K^) are located in the N‐terminal catalytic domain, two (*AARS* ^S627L^ and *AARS* ^E688G^) are located in the editing domain and the final two (*AARS* ^E778A^ and *AARS* ^D893N^) are located in the C‐Ala domain (Figure 3a and b). Of these variants, the *AARS* ^R329H^ variant was first discovered to segregate with CMT2N in two unrelated French families (Latour et al., 2010) and was then identified in a large Australian family (McLaughlin et al., 2012) and in a cohort of patients from one Irish and four British families (Bansagi et al., 2015), thus representing a recurrent variant worldwide. Yeast complementation assay results revealed impaired enzyme functions of the *AARS* ^R329H^ variant as well as the *AARS* ^N71Y^, *AARS* ^G102R^, *AARS* ^R326W^ and *AARS* ^S627L^ variants (McLaughlin et al., 2012; Motley et al., 2015; Weterman et al., 2018). In contrast, the cellular growth of yeast was not shown to be affected by the *AARS* ^E778A^ variant compared to that of the WT enzyme (McLaughlin et al., 2012), and the *AARS* ^R337K^ variant even improved yeast cell growth and showed a nearly 4‐fold increase in tRNA charging activity (Weterman et al., 2018). Interestingly, although different enzyme functions are caused by the *AARS* ^R326W^, *AARS* ^R337K^ and *AARS* ^S627L^ variants (Table 1), equivalent neural developmental toxicities were observed in the embryos of zebrafish after microinjections of human variant mRNAs, suggesting that the abnormal phenotypes in zebrafish are dominant‐negative or toxic gains of function (Weterman et al., 2018).

Other variants in *AARS*, including *AARS* ^D893N^ from a Chinese family (Zhao et al., 2012) and *AARS* ^E688G^ from an Irish family (Bansagi et al., 2015), were identified; however, their impacts on enzymatic activity and phenotypes in animal models need to be verified in the future.

As mentioned above, AlaRS includes an editing domain wherein two human CMT‐linked AlaRS variants (*AARS* ^S627L^ and *AARS* ^E688G^) are located (Figure 3a and b). In mice, a missense variant at A734E in the editing domain of murine AlaRS (*Aars* ^A734E^) can cause cell‐lethal accumulation of misfolded protein in neurons, leading to severe neurodegenerative phenotypes (Lee et al., 2006). Although the A734E variant in *Aars* is recessive, this finding, to some extent, pinpoints the fundamental roles of the editing activity of AlaRS for maintaining the accurate processing of genetic information and provides insight into novel mechanisms underlying human neurodegenerative diseases, such as AlaRS‐linked CMT.

Interestingly, the C‐Ala domain was once termed the dimerization domain because it provides contacts for dimerization in archaeal AlaRS (Naganuma et al., 2009). Over evolutionary time, the C‐Ala domain has been completely dispensable for aminoacylation but has developed distinct roles in higher organisms (Sun et al., 2016). Such a functional evolution of the C‐Ala domain allows AlaRS to be the single exception among the 19 other human aaRSs with no new motif or domain additions (Guo, Schimmel, et al., 2010). Perhaps for this reason, the *AARS* ^E778A^ variant in the C‐Ala domain shows WT‐like catalytic activity (McLaughlin et al., 2012), implying that nonenzymatic gain‐of‐function mechanisms underlie the pathogenesis of CMT2N.

## 5 *HARS* VARIANTS

**FIGURE 4:**
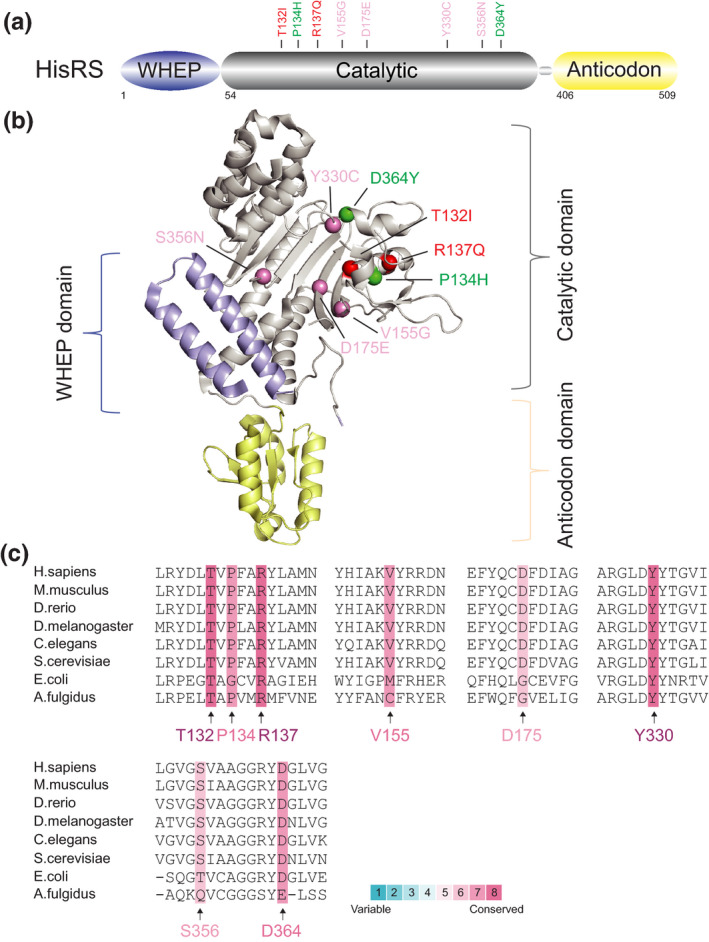
Distribution and conservation of CMT‐associated variant sites in human HisRS. (a) Functional domains of TyrRS including a WHEP domain (in purple), a catalytic domain (in grey) and an anticodon domain (in yellow). (b) The crystal structure of human HisRS (PDB entry 6O76). CMT variant sites in either schematic diagram (a) or crystal structure (b) are indicated with different colors based on enzymatic activity of each variant with the same priority ranking as Figure 1b. (c) Evolutionary conservation analysis of CMT‐linked HisRS across species. Sequence alignment of each variant site is indicated by the color intensity, with blue representing variable and red representing conserved

HisRS has a domain structure identical to that of GlyRS, as it consists of a WHEP domain, a catalytic domain and an anticodon binding domain (Figure 4a). *HARS* variants have been successively identified in dominant axonal CMT type 2W (CMT2W, OMIM# 616625) (Laura et al., 2019), with all variants being located in the catalytic domain (Figure 4a and b). Of eight variants, seven result in a loss of function as determined by yeast complementation assays, and neurotoxicity has been successfully recapitulated in transgenic *C. elegans* models of *HARS* ^R137Q^ and *HARS* ^D364Y^ variants (Abbott et al., 2018; Safka Brozkova et al., 2015; Vester et al., 2013). However, the discrepancies between yeast model phenotype and aminoacylation activities in vitro (e.g., the *HARS* ^D364Y^ variant is lethal in yeast cells but has normal charging activity in vitro) (Table 1), as well as the causal link between these variants and CMT2W, remain unclear.

Based on these considerations, a recent study further investigated charged tRNAs in actual patients with CMT using assays enabling the detection of aminoacylation within the human cell environment. Unexpectedly, no differences in the aminoacylation levels of tRNA^His^ and other tRNAs were observed between patients carrying the *HARS* ^P134H^ variant and their unaffected family members. Additionally, no correlation between the enzymatic activities of variants causing CMT2W and disease severity were found (Blocquel et al., 2019). Further biochemical and biophysical analyses of HisRS variants (*HARS* ^T132I^, *HARS* ^P134H^, *HARS* ^D175E^ and *HARS* ^D364Y^) demonstrated a conformational change that opens the dimerization interface of HisRS and likely exposes neomorphic surfaces that may mediate aberrant interactions with CMT2W‐causing variants. This work ruled out a correlation between enzymatic activity and disease severity, but instead showed a link between the degree of variant‐induced structural opening and disease severity, strongly supporting that *HARS*‐linked CMT disease is in fact not simply caused by a loss‐of‐function mechanism or a dominant‐negative effect of the variants but actually by open conformation‐driven gain‐of‐function mechanisms. The nonenzymatic functions gained by the *HARS* ^P134H^ and *HARS* ^D364Y^ variants in CMT are also rationalized by conservation analysis which showed highly conserved P134 and D364 sites with full enzymatic activity (Figure 4c).

It is worth noting that both GlyRS and HisRS contain a WHEP domain, which is indispensable for the neuronal toxicity caused by the *gars* ^P234KY^ variant in *Drosophila* (Grice et al., 2015). WHEP‐mediated suppression has also been shown to be a gain of function in the *GARS* ^G526R^ variant (He et al., 2011). In the case of HisRS, the conformational changes induced by HisRS variants strengthen the interactions between the WHEP domain and the C‐terminal part of the catalytic domain, which may help to open the dimerization interface (Blocquel et al., 2019). Interestingly, removal of the WHEP domain mostly had no effect on the aminoacylation activities of the WHEP‐containing aaRSs (Guo, Schimmel, et al., 2010), suggesting a potential relevance of the WHEP domain for CMT.

## 6 *WARS* VARIANTS

**FIGURE 5:**
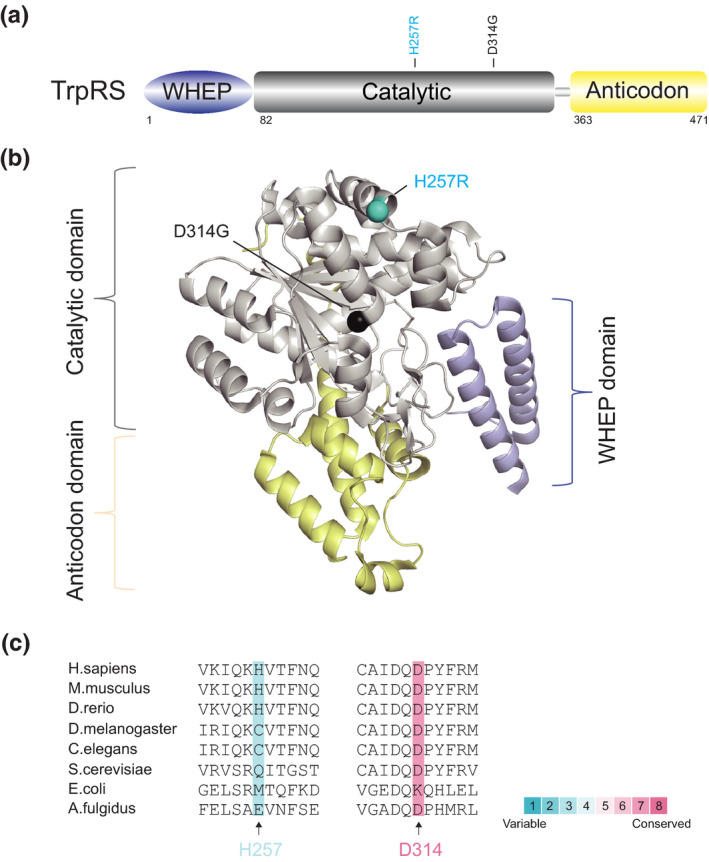
Distribution and conservation of CMT‐associated variant sites in human TrpRS. (a) Functional domains of TrpRS including a WHEP domain (in purple), a catalytic domain (in grey) and an anticodon domain (in yellow). (b) The crystal structure of human TrpRS (PDB entry 1R6T). CMT variant sites in either schematic diagram (a) or crystal structure (b) are indicated with different colors based on enzymatic activity of each variant with the same priority ranking as Figure 1b. (c) Evolutionary conservation analysis of CMT‐linked TrpRS across species. Sequence alignment of each variant site is indicated by the color intensity, with blue representing variable and red representing conserved

*WARS* encodes the human cytosolic TrpRS, which is composed of a WHEP domain, a catalytic domain and an anticodon binding domain (Figure 5a–c). *WARS* variants have just recently been identified in CMT, and the research on TrpRS in CMT is thus very limited.

The first missense variant of *WARS* (*WARS* ^H257R^) was identified in two unrelated Taiwanese pedigrees with autosomal‐dominant dHMN and later found in one additional dHMN family of European ancestry in Belgium (Tsai et al., 2017). This recurrent catalytic domain variant of *WARS* does not affect protein dimerization but does have a damaging and dominant‐negative effect on the enzymatic activity of TrpRS, resulting in disturbed protein synthesis and defective cell viability (Tsai et al., 2017). Furthermore, the variant, but not the WT, TrpRS inhibits neurite outgrowth in primary motor neurons and leads to neurite degeneration. Interestingly, in contrast to human WT TrpRS, which fails to complement *wrs1* deficiency in yeast models, the *WARS* ^H257R^ variant can partially complement *wrs1* deficiency in yeast cells, suggesting that the *WARS* ^H257R^ variant may change the structure of human TrpRS, enhancing the catalytic ability of the TrpRS variant toward yeast tRNA. Notably, the variant proteins exhibit enabled and enhanced binding to vascular endothelial‐cadherin (VE‐cadherin), leading to an enhanced angiostatic effect. This result is very similar to the previous finding that an aberrant GlyRS‐Nrp1 interaction interferes with the binding of VEGF, thus causing motor defects (He et al., 2015). In this regard, axonal neuropathy is very likely to be closely associated with the angiogenesis pathway, but this nonenzymatic function needs to be further investigated.

Very recently, another novel heterozygous *WARS* variant (*WARS* ^D314G^) was reported in a Chinese family presenting with a mild‐to‐moderate and late‐onset phenotype of dHMN (Wang et al., 2019). Further structural model analysis predicted that this catalytic domain variant might have an impact on the recognition, binding and activation of tryptophan; however, other functional assays are lacking.

## 7 OTHER PUTATIVE NEUROPATHY‐ASSOCIATED VARIANTS

### 7.1 *MARS* variants

Unlike other CMT‐linked aaRSs, methionyl‐tRNA synthetase (MetRS) functions as a monomer and associates with the multi‐synthetase complex (MSC), which is anchored by the N‐terminal GST domain. MetRS also has a conserved class I catalytic domain and an anticodon domain, followed by a C‐terminal‐appended WHEP domain with an unclear function. Four *MARS* variants have thus far been linked to CMT type 2U (CMT2U, # OMIM 616280) (Table 1). Three variants are located in the anticodon binding domain, and one is in the catalytic domain. The monoallelic variant of R618C in the *MARS* (*MARS* ^R618C^) was first described in a family with two affected patients who presented with late‐onset CMT2U and one unaffected member (Gonzalez et al., 2013). The *MARS* ^R618C^ variant is nonfunctional in yeast models, suggesting a loss‐of‐function feature; however, the potential mechanisms and contributions of this variant in CMT are less well characterized (Gonzalez et al., 2013). Subsequently, the *MARS* ^P800T^ variant was identified in a Korean family with late‐onset CMT2U (Hyun et al., 2014), and then in another Japanese family with late‐onset CMT2U (Hirano et al., 2016) and a Korean family with CMT2U but with a relatively early onset (Nam et al., 2016), suggesting that this recurrent variant causes variability and diversity of the CMT phenotype. Recently, two novel, likely disease‐associated missense variants, *MARS* ^R737W^ and *MARS* ^A397T^, were reported in a 13‐year‐old girl with CMT2U (Sagi‐Dain et al., 2018) and in an 11‐year‐old girl with early‐onset CMT2U (Gillespie et al., 2019), respectively. These findings enrich the spectrum of the *MARS* variants in CMT2U, but disease‐associated function of the variants is unclear, and the human genetic evidence for *MARS* variants in CMT remains unequivocal.

### 7.2 *NARS* variants

In addition to CMT, aaRSs variants have been frequently implicated in other neuropathies. For example, biallelic *NARS* variants were identified in 7 affected patients with recessive microcephaly from three unrelated families (Wang et al., 2020). Another study described de novo dominant heterozygous and biallelic recessive variants in the *NARS* in 32 individuals from 21 families, presenting with multiple neurodevelopmental defects (Manole et al., 2020). Interestingly, functional data indicated that genotypes with dominant heterozygous *NARS* variants produce a toxic gain‐of‐function, whereas the homozygous recessive variants probably induce a partial loss of function (Manole et al., 2020; Wang et al., 2020). Although neuropathies other than CMT are outside the scope of this review, these studies do shed light on the complex pathogenic mechanisms of aaRSs in neuropathies.

## 8 SUMMARY AND PROSPECTS

Despite the identification of an increasing number of causative genes, CMT remains incurable. A more accurate classification and deeper understanding of CMT remain great challenges to scientists.

One major challenge in this field is understanding the pathogenic commonalities among different aaRS‐linked CMT subtypes. Some aaRS variants may cause CMT through a loss of function (either through haplo‐insufficiency or a dominant‐negative effect). Many CMT‐linked aaRS variant sites, such as D146, P244, ETAQ, G273 and I280 in *GARS* (Figure 1c), G41 in *YARS* (Figure 2c), G102 and R329 in *AARS* (Figure 3c), and T132, R137 and Y330 in *HARS* (Figure 4c), are evolutionarily highly conserved, suggesting their important roles, and variations in them do have loss‐of‐function properties in vitro or in yeast models (Table 1). In addition, phenotypes caused by recessive homozygous variants or those beyond CMT can be rescued by WT proteins in animal models (Achilli et al., 2009; Chihara et al., 2007; Seburn et al., 2006), suggesting that some variants result in undefined loss of function. Nevertheless, the specific mechanism by which aminoacylation deficiency causes peripheral neuropathy remains unknown.

Many lines of evidence have confirmed the toxic functions gained from aaRS variants, and this discovery may serve as a shared disease‐causing mechanism for aaRS‐associated CMT. For example, different CMT‐linked variants of GlyRS, HisRS and TyrRS lead to shared neomorphic structural opening which allows aberrant interactions with membrane receptors or intracellular proteins, thereby interfering with certain signaling pathways and trafficking in motor and sensory neurons (Blocquel et al., 2017, 2019; He et al., 2011, 2015). This shared property may facilitate the identification of new therapies by targeting these opened sites in all aaRS‐linked CMT subtypes. In addition, both CMT2D and DI‐CMTC models in *Drosophila* share common genetic modifiers with nuclear localization (Ermanoska et al., 2014). This finding implies that the nuclear involvement of CMT‐linked aaRSs, such as TyrRS (Bervoets et al., 2019), may very likely be another shared pathogenic mechanism, but this topic needs further exploration. Last, except for TyrRS and AlaRS, three CMT‐linked aaRSs (GlyRS, HisRS and TrpRS) and one putative CMT‐associated MetRS contain a specific appended WHEP domain, which mostly does not affect the enzymatic activities of their aaRSs (Guo, Schimmel, et al., 2010) but does show a close association with aaRS‐linked CMT (Blocquel et al., 2019; Grice et al., 2015; He et al., 2011), implying a potential shared pathogenic mechanism. Further studies on the specific role of the WHEP domain in CMT are of great interest. Nonetheless, we cannot rule out the possibility that both functional losses and functional gains are simultaneously involved in the pathogenic mechanisms in certain CMT forms, although the underlying factors remain unknown.

Furthermore, multiple aaRS variants have been identified in CMT, but only a few have been tested in animal models or patient cells. To date, the CMT‐causing GlyRS is the only one that has been successfully recapitulated in mouse models, while the functions of the other aaRS variants have been determined in only yeast strains, which are apparently insufficient to illustrate the true regulatory functions of aaRSs under physiological conditions. The discrepant aminoacylation levels of the *HARS* ^P134H^ variant in yeast models and CMT patients suggests that the pathogenic mechanisms caused by aaRS variants in CMT are highly context‐dependent. Furthermore, simple experimental models of aaRS variants established in flies, fish and worms also require re‐evaluation in mammalian animal models or patient cells. As such, detailed analysis of a higher model system will be critical for addressing this issue, and it will help to not only distinguish the contributions of each aaRS variant to CMT, but also to determine the pathogenic commonalities among different aaRS‐associated CMT subtypes to further advance therapeutic interventions.

Last but not least, neither ideal biomarkers nor therapeutic targets are currently available for slowly progressive CMT, although we do have some promising candidates to target in the diagnosis and treatment of CMT, such as Nrp‐1, TRIM28 and nuclear TyrRS. Deeply understanding why peripheral nerves are predominantly affected in aaRS‐linked CMT and how they work within the human cell environment will hopefully lay the foundation for the precise stratification and targeted treatment of CMT in the future.

## CONFLICTS OF INTEREST

The authors report no conflicts of interest.

## AUTHORS’ CONTRIBUTIONS

H.Z. and L.S. wrote the paper and made the figures. Z‐W.Z. and L.S. designed the review.
